# Access, utilization, and barriers to using malaria protection tools in migrants to Iran

**DOI:** 10.1186/s12889-022-13913-3

**Published:** 2022-08-25

**Authors:** Hassan Okati-Aliabad, Alireza Ansari-Moghaddam, Mahdi Mohammadi, Jalil Nejati, Mansour Ranjbar, Ahmad Raeisi, Goodarz Kolifarhood, Fariba Shahraki-Sanavi, Alireza Khorram

**Affiliations:** 1 Health Promotion Research Center, Zahedan University of Medical Sciences, Zahedan, Iran; 2 National Program for Malaria Control, Center of Disease Control & Prevention, Ministry of Health and Medical Education, Tehran, Iran

**Keywords:** Migrants, Malaria, Protection, Iran

## Abstract

**Background:**

Imported malaria cases could be considered one of the threats to malaria elimination. Therefore, increasing migrants’ access to malaria preventive measures can play an essential role in maintaining appropriate conditions and preventing malaria outbreaks. This study aimed to provide detailed information about access, utilization, and barriers to using malaria protection tools in migrants to Iran.

**Methods:**

This study was conducted in a vast region consisting of 4 provinces and 38 cities located in the south and southeast of the country. Study participants were migrants who moved to the study area in the past three months. A sample of 4163 migrants participated in the study. They were selected through a multi-stage sampling method to obtain a representative community sample. Data were collected through interviewer-administered questionnaires about participants’ socio-demographic specification, commuting characteristics, travel aim, access, ways of preparing, and reasons to use or not to use malaria protection tools. Quantitative and qualitative variables were described and analyzed finally.

**Results:**

The mean age of individuals was 28.6 ± 10.8, with a range of 3–88 years old. Migrants’ country of origin was Afghanistan (56.6%), Pakistan (38.4%), and Iran (5%). Most migrants (69.2%) did not have malaria protection tools while staying in Iran. Among those who procured the protection tools, 74% used long-lasting insecticidal nets (LLINs), 13.4% used mosquito repellent sticks and coil, and 12.7% did not use any tools. Respectively, lack of knowledge about where they can get LLINs, followed by being expensive, unavailability in the market, not cooperation of health officer, and no need to use were expressed as the causes for having no access. The main reasons for non-using the tools were lack of knowledge about their application, followed by a defect in protection tools, ineffectiveness, and being harmful, respectively. Migrants who were supported by an employer accessed more to LLINs.

**Conclusions:**

This study reveals significant shortcomings in knowledge, access, and utilization of malaria protection tools among migrants in Iran. Inequitable access to public health services is predictable during migration; however, access to sustainable protection tools is recommended.

## Background

Despite malaria case incidence reduction in recent years, it is still considered a major global health challenge. Some countries have started the malaria elimination program to interrupt malaria transmission and finally, no indigenous cases. Focusing on malaria protection tools is one of the most critical points in this program. In the meantime, the imported case can cause the goal not to be achieved [1].

Iran, involved with the malaria elimination program, has reduced its indigenous cases to zero in 2018 and 2019. The policy of this program is based on three major strategies including further access to immediate and effective malaria treatment; more access to preventive services specially integrated vector management; and strengthening the malaria surveillance system. It focuses on interrupting the local transmission as the ultimate goal of malaria elimination by 2025. In addition, preventing and controlling imported cases that can cause the transmission to local residents is considered a critical issue. In contrast, the eastern neighbors, Afghanistan and Pakistan, are affected by malaria where the elimination program is not implemented [2, 3]. Afghanistan’s case incidence was higher in 2020 than 2015. Although Pakistan reported a decline in the mentioned years, it was less than 40% and insignificant. The total results of positive microscopy and rapid diagnostic tests (RDT) for Pakistan in 2015 and 2019 were 307,326 and 413,533, respectively. It was 119,859 and 173,860 for Afghanistan. However, there was no consistent decreasing trend in the number of cases in neighboring countries in the last decade [1].

Iran is one of the countries with the highest number of migrants globally [4]. So an investigation on the health challenges of migrants can be considered as an essential issue. It has resulted previously; most malaria cases in this country were attributed to Sistan and Baluchestan Province, with the highest number of malaria cases and a long shared border with Afghanistan and Pakistan. Most of them were imported cases, the citizens of Pakistan and then Afghanistan [3]. This province is a destination for international temporary migrants from those neighboring countries annually. The southern provinces, Kerman, Hormozgan, and Bushehr, with the lower malaria incidence than the southeastern areas, are considered the next destination [5]. The main motivations for migrants are seeking job opportunities, trade, visiting friends and relatives, attending traditional or cultural ceremonies, and going on a pilgrimage [6]. Most of them work as construction laborers or farmworkers [7]. The border areas of these countries with Iran have the high relative risk of malaria. The immigrants to Iran can be infected in this area, although their origin may be non-endemic [8]. There is a concern for introduced and indigenous cases followed by the imported cases in this part of the country [9]. Some studies showed that imported malaria cases could be considered one of the threats that can turn the foci from clear up to residual active [10, 11].

Therefore, increasing migrants’ access to malaria protection tools can play an essential role in maintaining appropriate conditions and preventing malaria outbreaks [12].

There is little documentation on migrants’ health challenges in Iran, and less attention has been paid to this crucial issue. This study was aimed to provide detailed information about the access, utilization, and barriers of using malaria protection tools in migrants to the country.

## Methods

### Study area

**Fig. 1:**
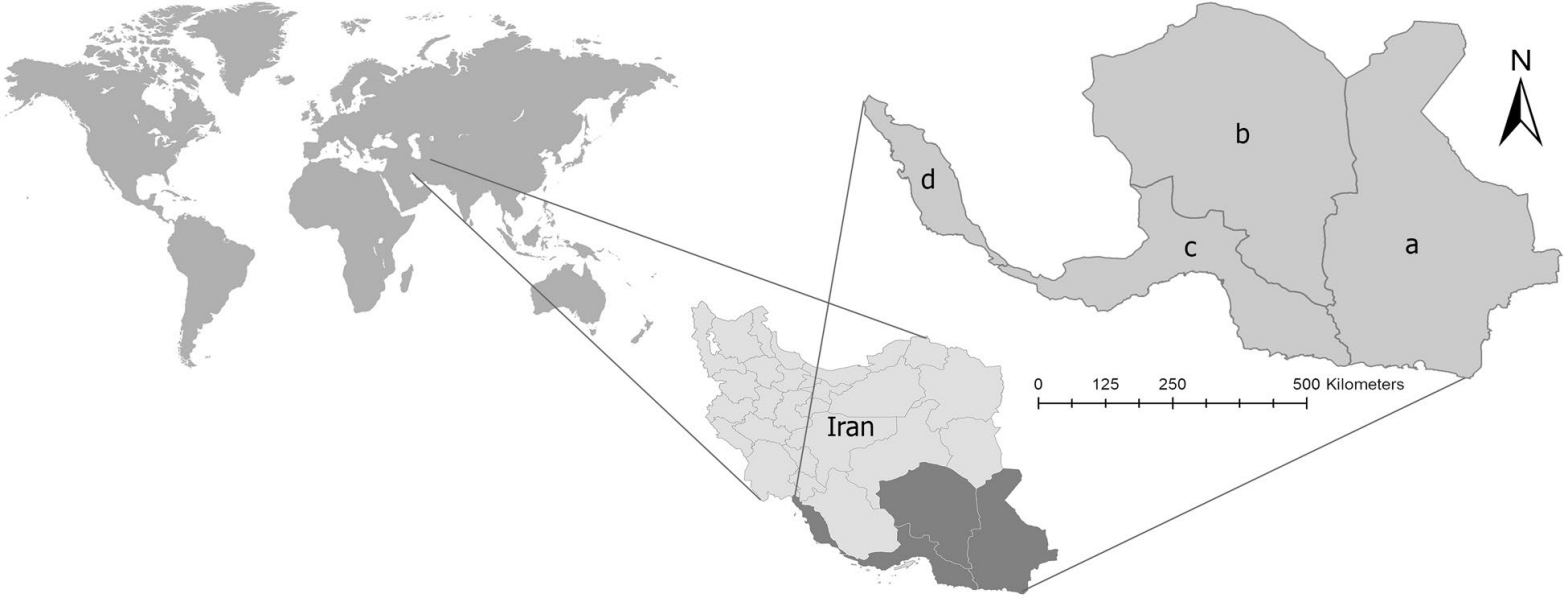
Location of study area, four provinces in southern and southeastern Iran; **a** Sistan and Baluchestan, **b** Kerman, **c** Hormozgan, **d** Bushehr

This cross-sectional study was conducted from April to September 2019. This investigation was done in a vast region of 4 provinces and 38 cities, located in the south and southeast of Iran (Fig. 1). The provinces were Sistan and Baluchestan (Lat: 25.09° N to 31.44° N; Lon: 58.78° E to 63.26° E), Kerman (Lat: 26.03° N to 32.04° N; Lon: 54.49° E to 59.48° E), Hormozgan (Lat: 25.23° N to 28.97° N; Lon: 52.41° E to 59.15° E), and Bushehr (Lat: 27.39° N to 30.25° N; Lon: 50.13° E to 52.96° E) [13–16].

Numerous studies have shown that these areas are suitable for mosquitoes breeding due to the favorable climate and monsoon currents, especially in the southeastern parts [17, 18]. In addition, five of the seven malaria vectors in Iran have been established and can be collected in these areas [12].

The study areas were selected based on published papers on the high risk of malaria and increased migration. In addition, undocumented evidence through interviews with local malaria experts was used.

### Study participants and sampling

Study participants were migrants who moved to the study area in the past three months for temporary farming, construction and service works, fishing, aquaculture, etc. A sample of 4163 migrants participated in the study. They were selected through a multi-stage sampling method to obtain a representative community sample. In the first stage, we used purposive sampling to include counties in each province with significant numbers of migrants. At the second stage, the main areas for the residence of the migrants in each county and the number of migrants in each area were identified. At the last step, the areas and the number of people in each region were selected proportional to the size and a simple random sampling method.

Inclusion criteria were migration to Iran in the last three months and informed consent to participate in the study. Individuals with severe physical or mental illness who could not participate were excluded from the study.

### Data collection

Data were collected through interviewer-administered questionnaires. The interviewers were local health workers who speak and understand Persian and Urdu languages. Data collection tools were developed after reviewing the relevant literature and meetings with malaria experts at the Center for Communicable Disease Control in the Ministry of Health and Medical Education of Iran and malaria experts at the Zahedan University of Medical Sciences. Data were collected on participants’ socio-demographic specification, commuting characteristics, travel aim, access, ways of preparing, and reasons to use or not to use malaria protection tools.

The research team held a briefing session and a training workshop for interviewers in all provinces. Training content included stating the purpose of the study, explaining the different parts of the questionnaire, the role of the interviewer and the supervisor, how to sample the study sites and participants, how to communicate with participants, seeking informed consent, interview techniques, the confidentiality of information, checking and sending completed questionnaires.

### Data analysis

Data were statistically analysed using Statistical Package for Social Science version 24 software (IBM SPSS Statistics for Windows). Quantitative and qualitative variables were described as mean ± standard deviation, number (percent), and odds ratio (OR), respectively. Furthermore, the chi-square test was used to determine the relationship between qualitative variables. Levels of significance were set at *P* < .05.

## Results

In total, 4163 migrants to Iran were investigated. The mean age of individuals was 28.6 ± 10.8, with a range of 3–88 years old. The majority of individuals were male (87.6%) and married (68.8%). Migrants’ country of origin was Afghanistan (56.6%), Pakistan (38.4%), and Iran (5%). The permanent living place of migrants was Afghanistan (61.3%), Pakistan (37.9%), and other countries (0.8%) (Table 1). Most of the Pakistani immigrants lived in Balochistan Province. Sindh and Punjab have had the highest number of immigrants since then. Panjgur, Kech (Turbat), Quetta, Kalat, Awaran, Kharan, and Khuzdar counties/districts had the highest number of immigrants. The majority of Afghan immigrants were from 4 provinces; Takhar, Kunduz, Kabul and Nimroz.

**Table 1:** Frequency distribution of demographic and commuting characteristics of migrants to Iran

Criteria	Item	n (%)
Age (year)	≤15	182 (4.4)
	16–20	867 (20.9)
	21–25	918 (22.1)
	26–30	835 (20.1)
	31–40	815 (19.6)
	More than 40	535 (12.9)
Gender	Male	3615 (87.6)
	Female (non-pregnant)	479 (11.6)
	Female (pregnant)	33 (0.8)
Marriage	Single	1282 (31.0)
	Married	2847 (68.8)
	Widow	11 (0.2)
Nationality	Iran	204 (5)
	Pakistan	1583 (38.4)
	Afghanistan	2335 (56.6)
Permanent living place	Pakistan	1491 (37.9)
	Afghanistan	2416 (61.3)
	Others	31 (0.8)
Number of times crossing the border in a year	1	2395 (61.7)
	2–3	1030 (26.5)
	> 3	458 (11.8)
Duration staying in Iran on the last trip (month)	1	1056 (29.2)
	2–3	862 (23.8)
	4–6	717 (19.8)
	> 6	981 (27.2)

Most migrants (61.7%) crossed the border once, and 11.8% commuted more than three times a year. About half of migrants planned to stay in Iran for less than four months and 27% more than six months. Afghan migrants crossed the border fewer times with more extended stays in Iran (Table 1).

Regardless of the type of employment, most working migrants were male, married, Afghani, aged 16–30 years, lived permanently in Afghanistan, and stayed temporarily in Iran. Most non-working migrants were Pakistani and 16–30 years old. The frequency distribution of demographic characteristics was significantly different among non-working, with, and without employer migrants (Table 2).

**Table 2:** Frequency distribution of travel aim in terms of demographic characteristics

Criteria	Item	Working trip		Non-working trip	***P***_ value
		With employer	Without employer		
Gender	Male	1878 (97.5)	1240 (88.9)	451 (60.1)	< 0.001
	Female	48 (2.5)	155 (11.1)	299 (39.9)	
Marriage	Single	681 (35.1)	416 (29.8)	168 (22.4)	< 0.001
	Married/widow	1256 (64.9)	982 (70.2)	573 (77.6)	
Nationality	Iranian	43 (2.2)	60 (4.3)	101 (13.4)	< 0.001
	Pakistani	764 (39.7)	430 (31.0)	380 (50.4)	
	Afghan	1118 (58.1)	897 (64.7)	273 (36.2)	
Permanent place	Pakistan	754 (39.9)	362 (27.5)	367 (56.9)	< 0.001
	Afghanestan	1137 (60.1)	954 (72.5)	278 (43.1)	
Type of stay	Permanent	715 (36.9)	632 (45.3)	307 (40.4)	< 0.001
	Temporary	1222 (63.1)	764 (54.7)	452 (59.6)	
Age (year)	<=15	49 (2.5)	62 (4.4)	67 (8.9)	< 0.001
	16–30	1386 (71.3)	864 (61.9)	334 (44.2)	
	31–50	471 (24.3)	396 (28.3)	275 (36.3)	
	> 50	36 (1.9)	76 (5.4)	80 (10.6)	

In Iran, 59.7% of migrants lived in a temporary place, and 50.8% had to change their homes more than once annually. Only 18% lived in a fully-finished building, while 40.1% lived in a shared room with their colleagues and 14.2% in a friend’s house. About 28% stayed in unequipped and inappropriate places.

Most migrants did not have access to malaria protection tools during staying in Iran (69.2%). Some migrants had access to long-lasting insecticidal nets (LLINs) (26%); mosquito repellent stick (3.4%), and coil (1.4%) (Table 3).

**Table 3:** Frequency distribution of access and use of malaria protection tools in migrants

Criteria	Item	N (%)	Pakistan	Afghanistan	OR (Af vs. Pa)
Malaria protection tools access in the current accommodation	LLINs	1074 (26.0)	525 (35.2)	475 (19.8)	0.45 (0.39, 0.52)
	MR stick	142 (3.4)	33 (2.2)	101 (4.2)	1.94 (1.30,2.88)
	MR coil	59 (1.4)	6 (0.4)	52 (2.2)	5.46 (2.34,12.75)
	None	2877 (69.2)	930 (62.2)	1772 (73.8)	0.59 (0.51,0.68)
Given access, ways of preparing malaria protection tools	Bought by the person	256 (20.6)	51 (9.5)	190 (31.6)	4.39 (3.14,6.14)
	Employer	59 (4.7)	29 (5.4)	29 (4.8)	0.89 (0.53,1.51)
	Health center	800 (64.4)	410 (76.5)	332 (55.1)	0.41 (0.32,0.52)
	Local people	23 (1.8)	8 (1.5)	15 (2.5)	1.70 (0.72,4.04)
	Traditional healers	2 (0.2)	0 (0.0)	2 (0.3)	–
	Friend’s gift	18 (1.4)	13 (2.4)	5 (0.8)	0.34 (0.12,0.96)
	Neighbor’s gift	5 (0.4)	3 (0.6)	2 (0.3)	0.60 (0.10,3.59)
	Others	28 (2.3)	–	–	–
	A combination of items	53 (4.2)	22 (4.1)	28 (4.6)	–
Given access, malaria protection tools used in the accommodation	LLINs	929 (73.9)	480 (87.1)	392 (62.7)	0.27 (0.20,0.36)
	MR stick	116 (9.2)	17 (3.1)	91 (14.6)	5.43 (3.19,9.25)
	MR coil	53 (4.2)	4 (0.7)	49 (7.9)	11.80 (4.23,32.92)
	None	160 (12.7)	50 (9.1)	93 (14.8)	1.77 (1.23,2.55)

Most migrants procured the protection tools either through the health care system (64.4%) or by buying personally (20.6%) (Table 3).

The using status among migrants who could prepare the protection tools was 73.9% LLINs, 13.4% MR stick and coils, and 12.7% did not use any tools (Table 3).

The reasons for non-using the tools were as follows; lack of knowledge about applying the tools (21.2%), defects in protection tools (16.2%), ineffectiveness (9.1%), and being harmful (2.8%). Migrants’ motives for lack of using protection tools were different (Table 4).

**Table 4:** Frequency distribution of non-access and no use of malaria protection tools in migrants

Criteria	Item	N (%)	Pakistan	Afghanistan	OR (Af vs. Pa)
Given not using, reasons not to use malaria protection tools in the current accommodation	Ineffective in preventing infection	112 (9.1)	73 (13.1)	37 (6.2)	0.44 (0.29,0.66)
	Do not know how to apply the tools	261 (21.2)	135 (24.2)	120 (20.1)	0.79 (0.60,1.04)
	Harmful	35 (2.8)	9 (1.6)	26 (4.4)	2.78 (1.29,5.98)
	Defect in protection tools	200 (16.2)	119 (21.3)	72 (12.1)	0.51 (0.37,0.70)
Reasons for not accessing malaria protection tools during staying in the current accommodation	I do not know where to get malaria protection tools	2190 (57.2)	763 (54.0)	1362 (62.3)	1.41 (1.23, 1.61)
	Not available in the market	297 (7.8)	135 (9.6)	158 (7.2)	0.74 (0.58,0.94)
	Expensive, I cannot afford to buy	545 (14.2)	242 (17.1)	280 (12.8)	0.71 (0.59,0.86)
	I asked form health system but the health officer did not give it to me	225 (5.9)	106 (7.5)	85 (3.9)	0.50 (0.37,0.67)

The reasons for having no access were as follows; they did not know where to get the tools (57.2%), unavailability in the market (7.8%), being expensive (14.2%), lack of health officer cooperation (5.9%), and no need to use (0.6%) (Table 4).

Most migrants traveled to work in Iran (82.9%), of which 58.1% worked under the supervision of an employer. Lack of access to malaria protection tools was more in migrants without an employer (75%) than in other groups (*P* < 0.001). Generally, access to MR stick or coil was low. Migrants who supported with an employer accessed more to LLINs (34.1%) compared to those with no employer (18.4%) and non-working migrants (19.9%) (*P* < 0.001). About one-third of migrants without an employer and non-working ones had procured the tools themselves compared to 11.4% of employed migrants (*P* < 0.001). About the reasons for not using the protection tools, 25.2% of employed workers and 12.5% of workers without employers reported that they did not know how to apply these tools (*P* < 0.001). More than half of migrants did not know where to get malaria protection tools (*P* = 0.545) (Tables 5 and 6).

**Table 5:** Frequency distribution of aim of travel in terms of access and use of malaria protection tools

Criteria	Item	working trip		Non-working trip	***P***_ value
		With employer	Without employer		
Malaria protection tools access in the current accommodation	LLINs	651 (34.1)	260 (18.4)	157 (19.9)	< 0.001
	MR stick	33 (1.7)	61 (4.3)	46 (5.8)	< 0.001
	MR coil	8 (0.4)	33 (2.3)	18 (2.4)	< 0.001
	None	1219 (63.8)	1056 (75.0)	568 (71.9)	< 0.001
Given access, ways of preparing malaria protection tools	Bought by the person	81 (11.4)	106 (31.5)	68 (35.8)	< 0.001
	Employer	49 (6.9)	–	–	–
	Health center	536 (75.4)	175 (52.1)	87 (45.8)	< 0.001
	Local people	3 (0.4)	15 (4.5)	5 (2.6)	< 0.001
	Traditional healers	0 (0.0)	1 (0.3)	0 (0.0)	0.261
	Friend’s gift	8 (1.1)	2 (0.6)	8 (4.2)	0.002
	Neighbor’s gift	2 (0.3)	2 (0.6)	1 (0.5)	0.726
	Others	15 (2.1)	8 (2.4)	4 (2.1)	
	A combination of items	17 (2.4)	27 (8.0)	17 (9.0)	< 0.001
Given access, malaria protection tools used in the accommodation	LLINs	566 (83.7)	224 (65.7)	135 (70.7)	< 0.001
	MR stick	18 (2.6)	49 (14.4)	47 (24.7)	< 0.001
	MR coil	7 (1.0)	33 (9.7)	13 (6.8)	< 0.001
	None	86 (12.7)	47 (13.7)	25 (13.2)	0.714

**Table 6:** Frequency distribution of aim of travel in terms of non- access and no use of malaria protection tools

Criteria	Item	working trip		Non-working trip	***P***_ value
		With employer	Without employer		
Given not using, reasons not to use malaria protection tools in the current accommodation	Ineffective in preventing infection	91 (12.7)	16 (4.9)	5 (2.7)	< 0.001
	Do not know how to apply the tools	180 (25.2)	41 (12.5)	38 (20.9)	< 0.001
	Harmful	10 (1.4)	20 (6.1)	5 (2.7)	< 0.001
	Defect in protection tools	114 (16.0)	54 (16.5)	32 (17.6)	0.868
Reasons for not accessing malaria protection tools during staying in the current accommodation	I do not know where to get malaria protection tools	1044 (56.9)	701 (56.6)	427 (59.0)	0.545
	Not available in the market	157 (8.6)	79 (6.4)	59 (8.1)	0.080
	Expensive, I cannot afford to buy	202 (11.0)	239 (19.3)	99 (13.7)	< 0.001
	I asked form health system but the health officer did not give it to me	96 (5.2)	85 (6.9)	44 (6.1)	0.168

## Discussion

Unlike previous, most migrants were Afghans in the present study, and some of them entered Iran via Pakistan. It was documented that many Afghan migrants pass through Pakistani malarious areas. Most Pakistani migrants to Iran are from high-risk malaria areas with poor welfare and care services near the shared border [19]. Therefore, their access to malaria protection tools is vital to maintaining the community’s health [20].

Social, cultural, linguistic, and religious ties between Afghanistan and Pakistan and Iran, especially in the areas close to the border, and one million registered and 1.5 million illegal Afghan migrants in Iran, lead to close relationship and mobility of people crossing the border [21]. These reasons can lead to their frequent cross-border traffic throughout the year. Some migrants had traveled more than three times a year in the present study. This migratory movement to malarious areas will increase the possibility of their infection and the occurrence of introduced cases in the country [22].

This study showed that most migrants have traveled to Iran for work. In many studies, it has been cited as the main reason for migration. Due to migrants working in malaria-endemic areas and their lack of knowledge about its transmission and protection usually have a higher prevalence of malaria than the resident population [23]. Creating the proper infrastructure to provide health services to migrants is essential for their health and the host country’s people.

Most migrants lived in temporary accommodation in the current study, and about 28% of them lived in unequipped and inappropriate places. Although this situation has been reported for migrants in some other countries, its improvement can play an essential role in preventing malaria outbreaks [24, 25]. Numerous studies have shown that access to suitable accommodation with welfare amenities such as electricity, air conditioner, etc., especially at night, can reduce mosquito densities and malaria transmission [18, 26].

In the meantime, access to malaria protection tools is even more critical. Unfortunately, the current study found that most migrants have no access to them. Although there is a policy of distributing free LLINs in the malaria elimination program in Iran [27], this protection tools do not cover all areas. However, migrants who had access to malaria protection tools; stated that they received it from the health system. In our study, Afghans had more access to MR stick and coil than Pakistani. At the same time, they were more likely to buy malaria protection tools. Perhaps the reason is the possibility of more accessible verbal communication and cultural match that facilitate the fulfillment of daily needs [28].

Using LLINs as one of the most critical malaria protection tools is recommended by the World Health Organization. Extensive LLINs in a malarious area can significantly reduce malaria [29]. Our study showed that LLINs were the main malaria protection tools among migrants, although it was not easily accessible. Similarly, it has been documented as the primary protection tool among migrants in some countries [30].

In the present study, insufficient knowledge about the protection tools led to non-use. This issue has also been reported in several studies [24]. A study conducted in Ethiopia resulted in education significantly associated with the knowledge and practice of malaria protection tools. This result can further highlight the role of health education in preventing malaria outbreaks caused by migrants’ traffic [31]. Although health education includes how to use malaria protection tools, the ways to provide them should also be informed. The current investigation showed some migrants did not know where they could get them. Raising awareness of migrant families about appropriate malaria prevention services has been suggested as essential in primary health care. In a study in the United Kingdom, practitioners and health workers highlighted providing tailored messages on preventing malaria that could have a considerable impact on malaria [32]. Generally, the elimination program should address equitable access to malaria preventive measures [33].

According to the present study results, access to malaria protection tools was higher among migrants who have employers that can be considered a capacity to promote the health of migrants. For instance, distribution of educational media among them can be done by employers. An investigation on Asian migrants to Angola showed most of them had been received information on malaria and its chemoprophylaxis from their employers. Even migrants with febrile illness prefer to seek care through their employer than in the public health system [34].

In our study, access to MR stick or coil was less. Actually; they are not free tools. Although some of them, like diethyl-3-methylbenzamide (DEET) used in national research, no document was found on their free distribution by the health system [35]. A study conducted on migrant populations in Myanmar showed N,N-diethyl-benzamide as MR stick has an influential role in reducing the incidence of *P. falciparum* and *P. vivax* infections. Inequitable access to public health services is predictable during migration; however, access to appropriate protection tool is considered a universal approach [36].

Migrants with an employer had more access to LLINs as a critical tool to prevent malaria. It seems employers have been involved in informing the health system to provide free health services. In other words, due to the lack of health system information on the situation of migrants without an employer, they have been forced to buy other tools instead of free LLINs, such as MR stick. Similarly, unregistered migrants were seldom achieved by LLIN-distribution campaigns in Cambodia. Actually, they were uninformed of the village malaria workers system due to poor social integration [20]. In this status, it is recommended to use the capacity of health volunteers. The experience conducted in Iran showed that volunteers play an essential role in providing health services to the people. More than 3700 trained health volunteers were used to perform rapid diagnostic tests (RDT) among unauthorized refugees. This capacity has been considered a significant advance towards eliminating malaria in Iran [7].

## Conclusions

This study reveals significant shortcomings in knowledge, access, and utilization of malaria protection tools among migrants in Iran. We recommend qualitative research for a better understanding of this issue. The enhanced awareness campaigns and planning to increase the migrant’s access to the malaria protection tools are also proposed. Strengthening migrants and employers’ awareness and capacity building to facilitate access to those tools will help eliminate malaria.

## Data Availability

The datasets used and/or analyzed during the current study are available from the corresponding author on reasonable request.

## Abbreviations

LLINs: Long-lasting insecticidal nets
MR: Mosquito repellent
